# Common variants in the *CPT1A* gene are associated with cataracts in Northern breeds of domestic dog

**DOI:** 10.1371/journal.pone.0320878

**Published:** 2025-04-04

**Authors:** Sally L. Ricketts, Saija Ahonen, Louise Pettitt, Jamie Freyer, Stuart Ellis, Christopher A. Jenkins, Maria Kaukonen, Mike Boursnell, Ellen Schofield, Oliver P. Forman, Hannes Lohi, Cathryn S. Mellersh

**Affiliations:** 1 Canine Genetics Centre, Department of Veterinary Medicine, University of Cambridge, Cambridge, United Kingdom; 2 Department of Veterinary Biosciences, Faculty of Veterinary Medicine, University of Helsinki, Helsinki, Finland; 3 Department of Medical and Clinical Genetics, Faculty of Medicine, University of Helsinki, Helsinki, Finland; 4 The Folkhälsan Research Center, Helsinki, Finland; 5 Wisdom Panel, Mars Petcare Science and Diagnostics, Portland, Oregon, United States of America; 6 SRE Testing, Preston, Lancashire, United Kingdom; 7 Wisdom Panel, Mars Petcare Science and Diagnostics, Leicestershire, United Kingdom; Long Island University - CW Post Campus: Long Island University, UNITED STATES OF AMERICA

## Abstract

Primary hereditary cataract affects many purebred domestic dog breeds and is a major cause of visual impairment in dogs. Cataracts are common in Northern breeds such as the Siberian Husky, Alaskan Malamute and Samoyed, but their aetiology is currently unknown. Only two genetic loci are known to be causally related to primary hereditary cataracts in the dog. To search for genetic loci associated with cataracts in Northern breeds, we used a genome-wide association study approach in three breeds—Siberian Husky, Alaskan Malamute and Samoyed. Cases were defined as dogs with bilateral posterior polar subcapsular cataracts and controls were at least four years of age with no evidence of cataracts or other ocular abnormality. We found a genome-wide statistical association for cataracts in the Siberian Husky on canine chromosome 18 (P-value: 1.1 x 10^ − 7^), which was independently replicated in a second larger case-control set (P-value 9.8 x 10^ − 29^). The Samoyed breed also showed evidence for association in the same genomic region (P-value: 2.4 x 10^ − 5^). We subsequently used targeted resequencing of the associated region (6.5 Mb) in ten Siberian Huskies and whole genome sequencing of a Husky, Malamute, Samoyed and Norwegian Buhund case to conduct fine-mapping and screen for candidate causal variants. These analyses identified a region of linkage disequilibrium in the four breeds containing common variants in the carnitine palmitoyltransferase 1A (*CPT1A*) gene that are strongly associated with bilateral posterior polar subcapsular cataracts in the Siberian Husky, Samoyed, Icelandic Sheepdog and Norwegian Buhund and we demonstrate that *CPT1A* is expressed in the dog lens and retina through RNAseq. Our findings represent a novel locus for cataracts in dogs. However, further work is needed to elucidate the pathophysiology underlying the association between *CPT1A* and cataracts in Northern breeds.

## Introduction

Primary hereditary cataract (HC) affects nearly 100 purebred dog breeds and is a major cause of blindness and visual impairment in dogs [1,2]. Cataracts are phenotypically diverse and can develop in dogs for several reasons, including genetic defects, localised inflammation, congenital disorders, metabolic or nutritional disturbances, trauma, or age-related changes in the lens. Primary hereditary forms tend to exhibit marked breed specificity in ophthalmic appearance and position within the lens, age of onset, rate of progression and degree of bilateral symmetry. Currently, there are only two known causal loci for HC in the dog—the *FYCO1* gene, which is mutated in Wirehaired Pointing Griffon Dogs with juvenile cataract [3], and the *HSF4* gene, which is relevant to Australian Shepherds, Boston Terriers, French Bulldogs and Staffordshire Bull Terriers [4–6].

**Fig 1:**
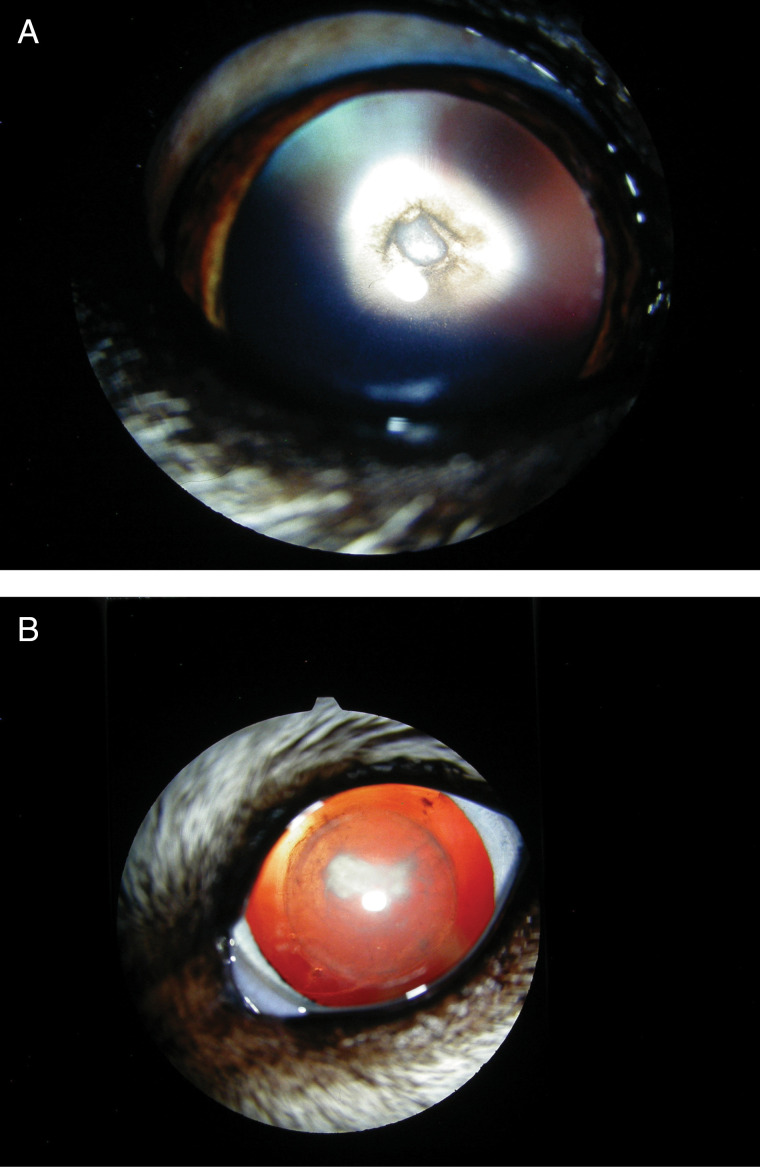
Example of cataracts in the Siberian Husky. (A) A three-year-old Husky with a typical PPSC showing a dense triangular polar opacity but also with radiating extensions of opacity along the posterior lens fibres; (B) A Husky first diagnosed with HC/PPSC at age 15 months. This image was obtained at age 9 years. Whilst the dog has nuclear sclerosis (a normal ageing change seen in dogs over 8 years of age), the cataract has also extended around the aged nucleus and cortical vacuoles are present.

In Northern or Arctic breeds, such as the Siberian Husky and Samoyed, a retrospective study of US dogs indicated that the prevalence of HC rose substantially over four decades between 1964 and 2003 from under 1% to up to 6% [7]. HC in these breeds typically presents as a bilateral cataract in the posterior polar subcapsular area of the lens (PPSC), although there is a distinct lack of published epidemiological/clinical studies. In the Siberian Husky, cataract development usually begins at around 9 months to 2 years of age and can in some cases progress both outward in a feathering pattern and forward into the cortex of the lens (Fig 1). This progression can limit vision, and in Alaskan Malamutes with visual impairment increased aggression has been anecdotally reported. The Husky has also been shown to be at a higher risk of retinal detachment pre- and post-cataract treatment [8,9]. The early age of onset for HC in the Husky suggests a strong genetic component to cataract development in this breed.

To identify genetic loci conferring risk of HC in Northern breeds, we conducted a genome-wide association study of HC in three individual breeds—the Siberian Husky and its closely related breed, the Alaskan Malamute, and the Samoyed. We identified a genomic region showing genome-wide statistical association with HC in the Siberian Husky, and used targeted resequencing and whole genome sequencing (WGS) to fine-map the region and identify potential causative variants. We tested potential candidate variants by genotyping additional Husky samples and other Northern breeds, and identified several variants that show strong statistical association with HC in the Siberian Husky, Samoyed, Icelandic Sheepdog and Norwegian Buhund.

## Results

### Genome-wide association study

**Fig 2:**
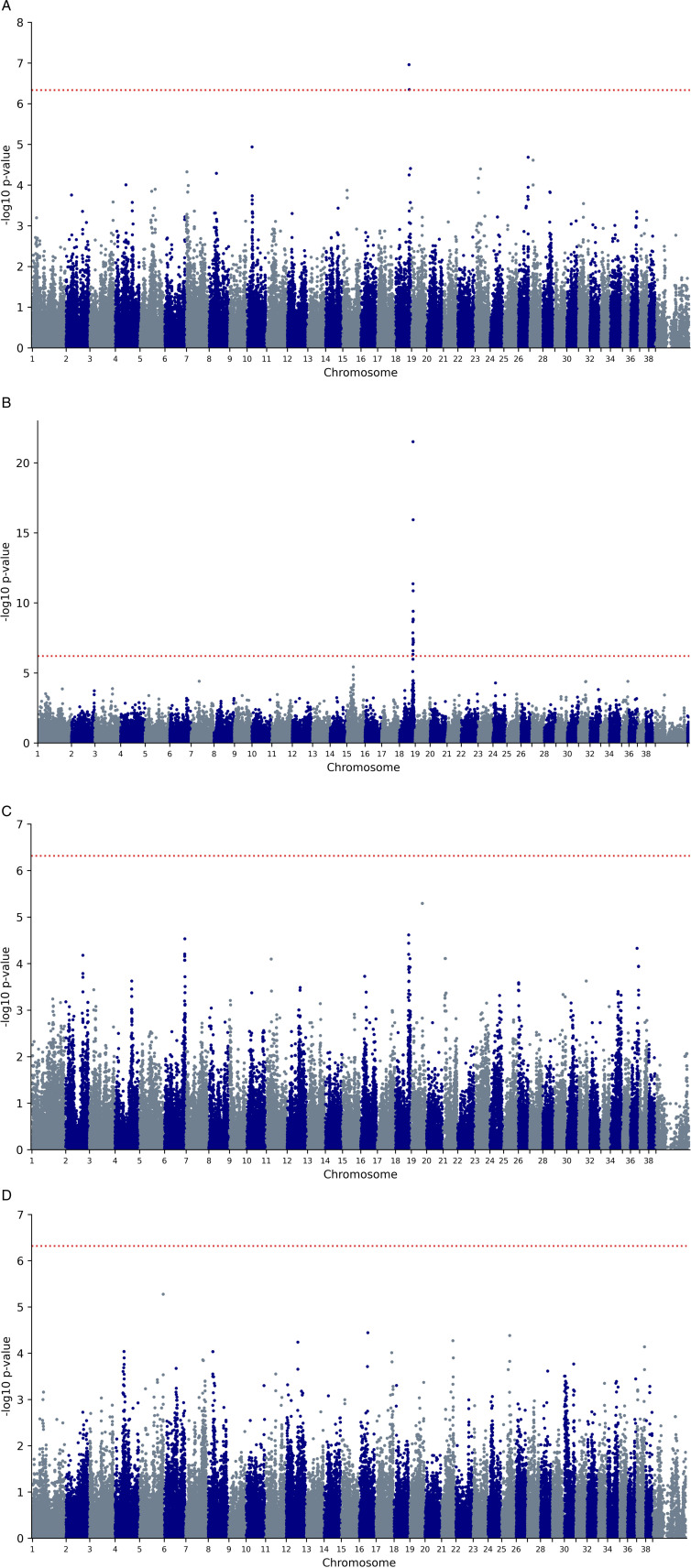
Manhattan plots depicting the results of GWAS analyses. (A) Siberian Husky (33 cases and 61 controls, 107,878 SNPs); (B) Siberian Husky replication set (152 cases and 149 controls, 81,230 SNPs); (C) Samoyed (23 cases and 25 controls, 103,719 SNPs); and (D) Alaskan Malamute (25 cases and 40 controls, 104,618 SNPs). Analyses for (A), (C) and (D) were conducted using a mixed model to adjust for overall population structure and relatedness within samples (see **Materials and methods**). For (B) analyses were conducted using GEMMA [10] to control for population substructure (see **Materials and methods**). The horizontal red dotted line denotes genome-wide statistical association using Bonferroni correction (P <  4.6 x 10^ − 7^ for (A), (C) and (D); and <  6.2 x 10^ − 7^ for (B)). Plots (A), (C) and (D) show BROADD2 data (Illumina canineHD). Plot (B) shows array data mapped to the CanFam 3.1 genome assembly. See S1 Table for LiftOver to other assemblies.

We obtained genome-wide single nucleotide polymorphism (SNP) data for three breeds (Siberian Husky 33 cases and 61 controls, Samoyed 23 cases and 25 controls, Alaskan Malamute 25 cases and 40 controls) and found a genome-wide statistical association for HC in the Siberian Husky on canine chromosome 18 (P-value for strongest SNP BICF2P1390488: 1.1 x 10^ − 7^) (Fig 2A). This association was later replicated in a large independent set of 152 Husky cases and 149 Husky controls genotyped on a different array (see Materials and Methods) (P-value for strongest SNP chr18_49165418 (CanFam 3.1): 3.0 x 10^ − 22^) (Fig 2B). For the Samoyed we also found evidence for an association in the same CFA18 region, although this did not reach genome-wide significance (P-value for strongest SNP BICF2P1077601: 2.4 x 10^ − 5^) (Fig 2C). No clear association signals were identified for the Alaskan Malamute (Fig 2D). We conducted a combined analysis of the Husky and Samoyed to attempt to refine the CFA18 association signal and assess the presence of possible additional shared HC associations in the two breeds. However, we did not identify a single SNP that reached genome-wide statistical association in this combined analysis (S1 Fig), suggesting that any shared variant conferring susceptibility to HC lies on a different haplotype background between the two breeds and is therefore being captured by different GWAS SNPs. We also conducted a secondary GWAS analysis in the Siberian Husky conditioning on the top SNP to search for additional HC associations in this breed, but we did not identify any additional regions (S2 Fig).

**Fig 3:**
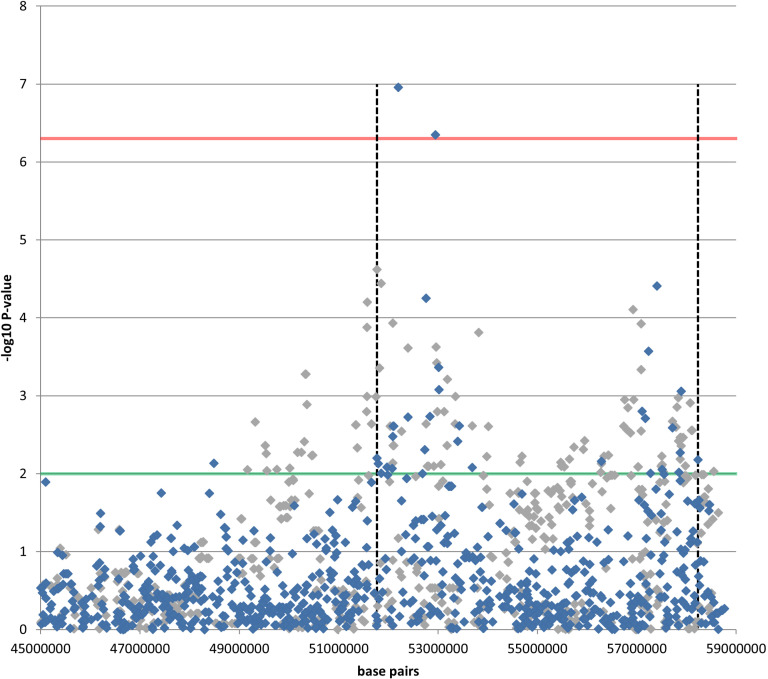
Partial CFA18 regional association plot of the genomic region surrounding the HC signal in the Siberian Husky (blue diamonds) and Samoyed (grey diamonds). The horizontal red dotted line denotes genome-wide statistical association (P <  4.6 x 10^ − 7^), and the green line represents the empirical statistical threshold used to delineate the critical region in the Siberian Husky (P-value < 0.01). The vertical black dashed lines denote the start and end points of this critical region at chr18:51773736 bp and 58230092 bp (BROADD2 genome build; see S1 Table for LiftOver to other canine assemblies). The full CFA18 regional association plot is given in S5 Fig.

Fig 3 shows the associated CFA18 region in detail for the Samoyed and Siberian Husky discovery sets. Analyses of the interrelationships among the top SNPs in the Siberian Husky (see **Materials and methods**) suggest that the region might comprise two independent association signals (Fig 3); however, it is possible that the sequence between harbours the susceptibility variant but because of lower linkage disequilibrium (LD) has not been well captured by the SNPs on the canineHD array. For the Samoyed and Alaskan Malamute, LD and homozygosity across the CFA18 region is higher (average r^2^ of SNPs = 0.22 and 0.18 respectively, Siberian Husky average r^2^ = 0.10). Analysis of the LD among the SNPs in the associated region in cases and controls separately did not refine the association signal in either the Siberian Husky or Samoyed—the top SNPs from both GWAS were not correlated (r^2^ > 0.3) with any nearby SNPs on the canineHD array. We therefore focused our efforts on further refining the association signal in the Siberian Husky and attempted to delineate a conservative critical region using an empirical statistical threshold of P ≤ 0.01 for SNP association results spanning the strongest SNPs in each sub-region. This resulted in a region of around 6.5 Mb (Fig 3).

### Targeted resequencing of the associated CFA18 region

We selected five bilateral PPSC cases and five controls (dogs over the age of 6 years with no evidence of cataracts or other ocular abnormality) for targeted resequencing of the 6.5 Mb delineated region (containing around 240 genes) in the Siberian Husky in order to search for a causative variant underlying the GWAS signal. Dogs were selected based on their genotypes for the two strongest GWAS SNPs in each sub-region as above (S2 Table). Following data analysis, we found 35,373 variants (4,569 in-dels and 30,804 SNPs) among the ten Siberian Huskies and the Boxer reference sequence (BROADD2 genome assembly). All variants showing perfect segregation with disease status through either a dominant or recessive mode of inheritance were taken forward (80 for the dominant model and 120 for the recessive model) and the data checked for errors by manually viewing each aligned variant in the Integrative Genomics Viewer (IGV) [11]. Variants where the putative risk allele was the same as the Boxer reference were excluded. This left 78 variants in or near 11 genes that were subsequently manually checked for gene annotation by multi-species alignments. Of these, ten SNPs were identified that were located in genic regions, including one nonsynonymous SNP in each of the *CPT1A*, *C11orf24*, *INCENP* and *MYRF* genes; one synonymous SNP in each of the *CPT1A*, *RCE1*, *INCENP* and *MYRF* genes; and two 3’UTR SNPs in the *RCE1* gene. We assessed the putative functional consequences of these ten SNPs using three *in-silico* methods—MutationTaster [12], SIFT [13] and PolyPhen [14]. The non-risk alleles at two nonsynonymous variants (chr18:52196958 (exon 11 of the *CPT1A* gene) and chr18:57736166 (exon 6 of the *MYRF* gene)) were conserved bases in > 25 eutherian mammals and the risk alleles predicted to be damaging to protein function—these SNPs were investigated further.

### Association analyses of candidate SNPs identified from targeted resequencing

We assessed the association of SNPs 52196958 and 57736166 with HC in our extended case-control sets for each Northern breed. We found that SNP_52196958 was strongly associated with bilateral PPSC in the Siberian Husky (Table 1). All but two of the 43 Siberian Huskies affected with bilateral PPSC were homozygous for the risk allele. However, this risk allele is common in our sample populations (66% frequency in the control group) and 40% of control dogs with clear eyes are homozygous for this allele (Table 1). For the Alaskan Malamute there was no strong statistical evidence for association with bilateral PPSC (Table 1) although the genotypes for this SNP showed a similar distribution among cases—38 of the 46 cases were homozygous for the risk allele and the remaining eight were heterozygous. The risk allele is more common in this breed—88% frequency in the control group (Table 1). In the Samoyed SNP_52196958 was also strongly associated with bilateral PPSC, although the genotype distributions in cases appear less compelling (Table 1). Interestingly, in the Samoyed, SNP_52196958 is highly correlated to the top SNP from the Samoyed GWAS (BICF2P1077601) (r^2^ = 0.84), but only partially to the top Siberian Husky GWAS SNP BICF2P1390488 (r^2^ = 0.47). By contrast in the Siberian Husky, the top SNP in the Siberian Husky GWAS (BICF2P1390488) shows a very strong correlation with SNP_52196958 (r^2^ 0.94), but only partially to the top Samoyed GWAS SNP BICF2P1077601 (r^2^ 0.46). This might explain the differences in topology of the GWAS data between the two breeds (Fig 2) and the lack of a common associated SNP (S1 Fig).

**Table 1:** Association between SNP_52196958 and HC in Northern breeds.

				Genotypes^†^ (cases/controls)			Allele frequencies^†^ (cases/controls)		Fisher’s exact P-value
Breed	Case definition ^‡^	Control definition ^∞^	n cases/controls	GG	GC	CC	G	C	
Siberian Husky	OU PPSC	NAD	43/138	41/55	2/ 72	0/11	0.98/0.66	0.02/0.34	6.3 x 10^ − 11^
Siberian Husky	Other cataract	NAD	18/138	14/55	3/72	1/11	0.86/0.66	0.14/0.34	6.9 x 10^ − 3^
Samoyed	OU PPSC	NAD>=6 years of age	30/83	17/10	7/36	6/37	0.68/0.34	0.32/0.66	1.3 x 10^ − 5^
Samoyed	Other cataract	NAD>=6 years of age	13/83	3/10	4/36	6/37	0.38/0.34	0.62/0.66	0.46
Alaskan Malamute	OU PPSC	NAD	46/120	38/92	8/26	0/2	0.91/0.88	0.09/0.13	0.83
Alaskan Malamute	Other cataract	NAD	19/120	16/92	3/26	0/2	0.92/0.88	0.08/0.13	0.82
Icelandic Sheepdog	OU PPSC	NAD>=6 years of age	12/35	12/9	0/17	0/9	1.00/0.50	0.00/0.50	3.0 x 10^ − 5^
Norwegian Buhund	OU PPSC	NAD>=4 years of age	10/9	10/4	0/3	0/2	1.00/0.61	0.00/0.39	0.01
Finnish Lapphund	OU PPSC	NAD>=6 years of age	27/81	12/27	11/46	4/8	0.65/0.62	0.35/0.38	0.32
Lapponian Herder	OU PPSC	NAD>=6 years of age	15/68	10/49	2/18	3/1	0.73/0.85	0.27/0.15	0.03 *

‡ OU PPSC: bilateral posterior polar subcapsular cataract; Other cataract: unilateral PPSC, cataract atypical for breed, e.g., nuclear, cortical, punctate cataract.

∞ NAD: no abnormality detected.

† G =  risk allele; C =  non-risk allele (BROADD2 genome build. See S1 Table for LiftOver of co-ordinates amongst canine genome assemblies).

*reciprocal association.

We also conducted a secondary analysis in each breed to examine the association between SNP_52196958 and other types of cataract. This set of cases encompassed dogs with either unilateral PPSC or cataracts atypical for the breed (e.g., nuclear, cortical, or punctate cataracts). We found evidence for association in the Siberian Husky, but not in the Samoyed or Alaskan Malamute (Table 1).

SNP_57736166 did not show a strong association with HC in the Siberian Husky (P = 0.01) or Samoyed (P = 0.02) (S3 Table), and the non-risk allele was fixed in the Alaskan Malamute. It was therefore discarded from further investigation.

Icelandic Sheepdogs are an Arctic breed that also develops HC in the form of bilateral PPSC. We therefore genotyped the *CPT1A* SNP_52196958 in 12 cases and 35 controls and found an association with HC consistent with the Siberian Husky and Samoyed (P = 3.0 x 10^ − 5^) (Table 1). We also genotyped two other Finnish Northern breeds: Finnish Lapphunds (27 bilateral PPSC cases and 81 controls), which showed no statistical association (P = 0.32) and Lapponian Herders (15 bilateral PPSC cases and 68 controls), which showed a weak reciprocal association (P = 0.03) (Table 1).

To assess how common *CPT1A* SNP_52196958 is in other non-Arctic breeds, we genotyped the variant in a multi-breed panel of 96 dogs (up to three dogs from 32 breeds). All breeds were homozygous for the non-risk allele, with the exception of three breeds—the Flat Coated Retriever (one heterozygous and one homozygous for risk allele), Miniature Bull Terrier (one heterozygote), and Tibetan Spaniel (one heterozygote). These breeds have not been formally reported to develop hereditary cataracts, but a search of our sample database identified 11 Miniature Bull Terriers reported to be affected by cataracts (with variable phenotype). We obtained genotype data for SNP SNP_52196958 for 10 of these dogs; seven were homozygous for the non-risk allele and three were heterozygous (S4 Table), which does not follow the association pattern seen in the Northern breeds, although limited inference can be made due to the small size of the sample set. In addition we genotyped case-control sets for three other HC-affected (bilateral PPSC) breeds: Irish Red and White Setters (17 bilateral PPSC cases and 18 controls over the age of 6 years); all homozygous for the non-risk allele, Australian Shepherds (12 bilateral PPSC cases and 36 controls over the age of 8 years); all homozygous for the non-risk allele, and Golden Retrievers (46 bilateral PPSC cases and 45 controls over the age of 6 years); no association with HC in this breed (P = 0.5) (S4 Table).

As SNP_52196958 lacks the specificity to be predictive for HC despite being strongly associated with the condition in the Northern breeds, we returned to our resequencing results to examine additional lower-impact variants within the *CPT1A* gene that might be candidate causal variants for HC in these breeds. In total, 36 variants spanning the gene (33 SNPs and three 1 bp in-dels, including the top Husky GWAS SNP at BROADD2 chr18:52197069 bp) segregated with HC in our resequencing data. Of these, one was identified as a potential candidate as it is close to a splice site (BROADD2 chr18:52196196 bp). The synonymous *CPT1A* SNP described above (BROADD2 chr18:52193311 bp) was the only other variant in this gene in our data that was predicted to be functional. The remaining 34 variants were intronic. We therefore tested these two variants (SNP_52196196 and SNP_52193311) in our additional sample sets to assess the strength of their association with HC. Splice site SNP_52196196 showed a perfect correlation with SNP_52196958 in the Husky and Icelandic Sheepdog and was discordant in only one Alaskan Malamute and one Samoyed, making the association with HC marginally weaker than SNP_52196958 in the latter breed (S5 Table). In the Lapponian Herder the association was stronger than SNP_52196958 (S5 Table). Synonymous SNP_52193311 showed a weaker association with HC than SNP_52196958 in the Husky, Samoyed and Icelandic Sheepdog (S6 Table); by contrast the association between SNP_52193311 and HC in the Lapponian Herder was the strongest of all three SNPs (P = 3.3 x 10^ − 3^) (S6 Table). We subsequently genotyped a small set of 10 Norwegian Buhund bilateral PPSC cases and 9 controls for the most provocative nonsynonymous SNP_52196958 from the above analysis and again found an association with HC consistent with the Siberian Husky and Samoyed (P = 0.01) (Table 1). Further analysis of the 34 intronic variants above predicted that one resulted in the loss of a potential splice acceptor (SNP_52198711). We therefore genotyped a subset of our additional sample sets for this variant and it showed perfect correlation with SNP_52196958 in the Alaskan Malamute and Norwegian Buhund, with one discordant in the Husky and Icelandic Sheepdog making it less strongly associated than SNP_52196958 (S7 Table).

We also conducted a re-analysis of the targeted resequencing data allowing for one discordant case or control in our initial filtering step, and identified a nonsynonymous SNP in the pyruvate carboxylase (*PC*) gene that we genotyped in our expanded Husky and Malamute case-control sets. This did not show any association with HC (P-values for Fisher’s exact test 0.38 and 0.47, respectively) and so was not investigated further (S1 File).

### WGS analysis of the CFA18 region

As our targeted sequence data encompassing the HC-associated region was incomplete due to the masking of repetitive sequences, we subsequently generated whole genome sequences of bilateral PPSC cases (one of each of Alaskan Malamute, Siberian Husky, Samoyed and Norwegian Buhund) enabling assessment of more complete variant data across the associated region. From the original target sequencing data, we assessed LD amongst SNPs across the captured region in the 10 Huskies and defined a region of 28,711 bp spanning intron 3 to intron 16 of *CPT1A* (co-ordinates chr18:49660662–49689373 (CanFam 4 - see S1 Table for LiftOver to other canine genome assemblies) that was captured at an r^2^ of 1.0 by the top GWAS SNP BICF2P1390488. Across this region there were 39 variants shared amongst the five target sequenced Husky cases and the four WGS cases. The shared haplotype was shortened at the 5’ end to 21,718 bp if one of the variants that differed amongst these cases was used to further delineate the region (SNP_49667655, CanFam 4; see S8 Table for the complete list of 40 variants in the shared haplotype amongst five target sequenced Husky cases and four case WGS (Husky, Malamute, Norwegian Buhund and Samoyed). There were no additional variants in the WGS data in this region that were not present in the target data and a manual scan for structural variants across this region did not find any provocative variants present in all four WGS cases.

### cDNA analyses of CPT1A sequence in canine lens

To confirm that the *CPT1A* gene is expressed in ocular tissue in the dog and to verify the transcript and coding sequence of the gene, we obtained mRNA sequence from a healthy canine retina using mRNA-seq, and partial coding *CPT1A* mRNA sequence (exons 2–16) from a healthy canine lens. The RNA-seq and partial lens data confirmed that the dog retinal *CPT1A* transcript is identical to the published canine liver mRNA sequence (Genbank accession number NM_001286860.1) and that the predicted coding sequence in both BROADD2 and CanFam 3.1 genome builds was incorrect and omitted exon 1 (5’ untranslated region) of the gene, which has now been corrected in the CanFam 4 genome assembly. We also used the DoGA Expression Atlas [15] to examine the expression of *CPT1A* in this dataset, which further confirmed its expression in ocular tissue and retina (S3 Fig).

## Discussion

Our genome-wide association and targeted resequencing study of hereditary cataract in the Siberian Husky has identified SNPs in the *CPT1A* gene that are strongly associated with bilateral posterior polar subcapsular cataracts in four Northern breeds of domestic dog—the Siberian Husky, Samoyed, Icelandic Sheepdog and Norwegian Buhund. In our secondary analyses of the Siberian Husky and Samoyed we found only a weak or null association between the *CPT1A* variants and other types of cataract, respectively, providing evidence that the *CPT1A* association is limited to PPSC, which is the typical cataract that defines HC in these breeds. (The category ‘Other cataract’ in the Siberian Husky included some dogs with uniocular PPSC, which likely explains the weak association observed in this category (Table 1)). However, the risk alleles for HC are common in all of these breeds; and despite reasonable sensitivity (ability to correctly designate a case), their weak specificity (ability to correctly designate an unaffected dog) means that they are unsuitable for preventive DNA testing.

Our findings have some inconsistencies. Firstly, the *CPT1A* associations did not extend to the Alaskan Malamute, which was an unexpected finding as this breed is closely related to the Siberian Husky and clusters with it in genetic analyses of breed ancestry [16]. However, genotype proportions for the cases were directionally consistent with the other two breeds and the risk alleles were very common— ~ 88%. Secondly, there was no association with HC for the Finnish Lapphund and, by contrast, the association in the Lapponian Herder appeared reciprocal to the association seen in the Siberian Husky for one of the variants. As the non-risk alleles are the predominant alleles in the majority of the other non-Arctic breeds that we tested, this result could be due to chance given the case-control numbers tested. Moreover, these two breeds were reconstructed following a population bottleneck during World War II and do not have the ancient origins of the Husky, Malamute and Samoyed, and so may be genetically divergent from these breeds. However, the *CPT1A* variants are present in other breeds such as the Golden Retriever, a breed which also develops HC typified by bilateral PPSC, but no association with HC was observed in our sample set of 46 cases and 45 controls (S4 Table).

The above findings indicate that whilst we have identified a novel locus associated with HC in the dog, the associated locus is not singly causal for HC in these breeds. There are several possible scenarios. The risk alleles of the strongest associated *CPT1A* SNPs may be linked to a rarer causal variant that we have not identified through analysis of our resequencing data. Our GWAS data initially suggested the presence of a second independent sub-region; however, the most provocative functional SNP from this region was tested (SNP_57736166) and its association with HC was weaker than the top GWAS SNP from this sub-region (BICF2G630689379). From analyses of the LD among the variants we have studied, it appears likely that this second sub-region is weakly capturing the main susceptibility locus at *CPT1A* and it was not replicated by the independent GWAS (S4 Fig). Although we conducted a secondary GWAS analysis in the Husky to search for additional associated regions conditioning on the strongest GWAS SNP, with no clear signals it is possible that the Husky and related breeds are fixed for a second susceptibility variant elsewhere in the genome that is not present in other non-Arctic breeds. It could be that one of the *CPT1A* variants is the true susceptibility variant underlying this association and there is an environmental trigger for the development of HC that other breeds are not exposed to. Whilst we did not have the resources to genotype all of the variants in the shared *CPT1A* haplotype in our extended sample sets, we would have been unlikely to distinguish the candidate causal variant using statistical association. Clearly, along with functional expression assays in cellular studies to define which of the 39 variants in this haplotype has a functional effect on the *CPT1A* protein in ocular tissue, more substantial genetic and epidemiological studies are needed to examine the presence of additional risk factors for HC in these breeds.

Despite being unable to confirm a causal variant underlying the genetic mapping data, we have fine-mapped our association to within the *CPT1A* gene. The association between cataracts and the *CPT1A* gene is intriguing and has not previously been reported for any species. The *CPT1A* gene encodes carnitine palmitoyltransferase 1A, an outer mitochondrial membrane protein that is predominantly expressed in the liver and is responsible for regulating long-chain fatty acid beta-oxidation. *CPT1A* has also been shown to be expressed in human and rat retinal pigment epithelium and other neuroretinal cell types, suggesting that this process is also part of retinal metabolism [17]. Deficiency of the CPT1A protein is associated with autosomal recessive inherited severe and fatal episodes of hypoketotic hypoglycaemia in infants after illness or fasting and is highly prevalent in many indigenous Arctic human populations [18]. It is thought that the deleterious alleles perpetuate in these populations due to an adaption to their traditional high-fat, high-protein diet [19] and it is suggested that the progressive retinopathy seen in these types of disorders might reflect an accumulation of toxins in the retina caused by the deficiency in this metabolic process [17]. It is interesting to speculate that the posterior polar subcapsular cataracts seen in the Northern breeds could be secondary to a subclinical metabolic defect that causes reactive mediators to be released into the vitreous humour from the retina [20]. We do not have direct functional evidence to examine how cataract susceptibility might be conferred by the variants at this locus as we did not have access to ocular material from either an HC-affected or unaffected Siberian Husky. However, we obtained coding mRNA sequence from a normal canine lens and retina, spanning the variants that we describe here, which demonstrate that *CPT1A* is also expressed in these tissues in the dog, supported by data from the DoGA expression atlas. Interestingly, a study of Huskies identified a genomic duplication approximately 4.2 Mb upstream of our top GWAS SNP that is associated with eye colour in the breed [21], however, this was not associated with HC in our Husky GWAS sample set (S9 Table).

Hereditary cataracts in dogs has previously been thought to be a simple Mendelian condition in several dog breeds, and in a few breeds this appears to be the case [3–6]. However, the paucity of cataract-associated mutations identified in the dog to date coupled with the results of our study indicate that this disorder may represent a complex disease, with perhaps multiple susceptibility alleles within a breed accompanied by environmental factors that are yet to be identified. This hampers efforts to develop diagnostic tools for prevention of this disorder in the Northern breeds, and much larger prospective studies are required to tease apart the aetiology of hereditary cataracts in these breeds.

In conclusion, we have identified variants in the *CPT1A* gene that are strongly associated with hereditary cataracts in the Siberian Husky, Samoyed, Icelandic Sheepdog and Norwegian Buhund breeds of domestic dog. However, these variants do not predict cataract development and are unsuitable for preventive DNA test development.

## Materials and methods

### Study samples

Samples were collected from privately owned pet dogs in the UK, Finland and overseas with their owner’s consent. DNA samples from dogs living in the UK were collected from buccal (cheek) swabs, or from the residual of blood samples drawn primarily as part of a veterinary procedure (Animal Health Trust Clinical Research Ethics Committee No. 24-2018E, University of Cambridge Department of Veterinary Medicine Ethics and Welfare Committee No. CR564). Elsewhere, we collected residual blood samples drawn as part of a veterinary procedure or buccal swab samples collected by owners or veterinarians, taken with their owner’s consent. In Finland, 3 ml EDTA blood samples were collected and archived in the dog DNA bank at the University of Helsinki with the owner’s consent and under the permission of the animal ethical committee of the County Administrative Board of Southern Finland (ESAVI/6054/04.10.03/2012). For the GWAS, we defined HC cases as dogs with bilateral PPSC. All cases were diagnosed by veterinary ophthalmologists. Controls were at least four years of age with no evidence of cataracts or other ocular abnormality after examination by veterinary ophthalmologists. Due to the modest sample numbers in this study, we sought to minimise potential misclassification (i.e., young dogs with clear eyes that could go on to develop HC and should thus have been classified as cases). We considered that this risk was small at this age cut-off, and it maximised the number of samples that we could include in our GWAS. (At least 70% of control dogs for the Alaskan Malamute and Siberian Husky were aged over 6 years, and for the Samoyed all control dogs were aged over 6 years.) Whole genome sequenced (WGS) dogs were bilateral PPSC cases of four breeds (Siberian Husky, Alaskan Malamute, Samoyed and Norwegian Buhund) that were homozygous for the risk allele G at SNP_52196958. The Siberian Husky WGS case was also part of the targeted sequencing set, described below.

For the Husky replication GWAS, samples were collected through routine commercial panel DNA testing. Genotype data was linked with Banfield Veterinary Hospital clinical records. Cases were defined as dogs diagnosed with either juvenile cataract or primary hereditary cataracts. Controls were dogs over the age of six and not diagnosed with any of the following: juvenile cataract, primary hereditary cataract, non-surgical cataract, surgical cataract. Genetic analyses were carried out on DNA extracted from owner-collected, non-invasive cheek swab samples or from blood/cheek swab samples collected at certified veterinary clinics in accordance with international standards for animal care and research. All dog owners provided consent for the use of their dog’s DNA sample for research purposes.

### Genome-wide SNP genotyping and analyses

Genomic DNA was extracted from blood or buccal swabs using standard protocols and normalised to 40–50 ng/µl for genome-wide SNP genotyping. We used the Illumina CanineHD array comprising 173,662 SNPs spanning the canine genome at a density of approximately 1 SNP per 14 kb. We obtained high-quality data for 25 cases and 40 controls for the Alaskan Malamute, 23 cases and 25 controls for the Samoyed, and 33 cases and 61 controls for the Siberian Husky. (Six dogs that had a sample call rate of <  95% were excluded.) SNPs were filtered based on minor allele frequency ( < 5% excluded) and call rate ( < 97% excluded) resulting in 104,618, 103,719 and 107,878 SNPs for analysis for the Alaskan Malamute, Samoyed and Siberian Husky, respectively.

We used the freely available software GWAS analysis package PLINK [22] to conduct basic unadjusted GWAS analyses independently within each breed. We also assessed for the presence of population substructure in our samples using multidimensional scaling in PLINK and corrected our data for any stratification using a mixed model [23]. These analyses were conducted in 2010. We defined genome-wide statistical association in the Siberian Husky as <  4.6 x 10^ − 7^; the Bonferroni correction for 107,878 SNPs. We assessed the interrelationships between the two top Husky SNPs using logistic regression and log-likelihood ratio tests as described in [24]. We conducted an unadjusted GWAS analysis in the Siberian Husky conditioning on the top SNP BICF2P1390488 using logistic regression in PLINK with SNP BICF2P1390488 genotype as a covariate in the regression model.

For the replication GWAS (conducted in 2022), genotyping was performed following manufacturer-suggested standard protocols on a custom 100K Illumina Infinium XT SNP microarray (Illumina, Inc., San Diego, CA, USA), at GeneSeek, Neogen Inc.. A total of 95,165 variants were available pre-QC. After the removal of dogs with >  5% missing SNP data; SNPs with a minor allele frequency ≤  1%; SNPs with >  5% missing data; and SNPs with an absolute difference in call rate between males and females >  2.5%; a total of 81,230 SNPs remained for analysis. GWAS analysis was performed using a linear mixed-model approach to adjust for population stratification using the software package GEMMA v0.98.5 [10]. A secondary analysis conditioning on top SNP 18_49165418 (CanFam 3.1 assembly; see S1 Table for LiftOver of co-ordinates amongst canine genome assemblies) was subsequently conducted in GEMMA using this SNP as a covariate.

Genomic inflation factors before and after correction for all GWAS analyses are given in S10 Table.

### Meta-analysis

We conducted a genome-wide meta-analysis of HC in the Siberian Husky and Samoyed discovery sets by combining summary statistics for each SNP from each breed (log odds ratios and standard errors) from unadjusted datasets. Analyses were done using STATA 9 (Stata 9. College Station, TX, USA). We used a fixed effects model and inverse-variance weighted averages of regression coefficients to obtain a combined estimate of the overall odds ratio for SNPs that passed quality control filters within individual breed sets and were common to both breeds. The genomic inflation factor for the meta-analysis is given in S10 Table.

### Resequencing and data analysis

We defined the critical region of association for HC in the Siberian Husky to span 51773736–58230092 bp on CFA18 – approximately 6.5 Mb (BROADD2 co-ordinates; see S1 Table for LiftOver of co-ordinates amongst canine genome assemblies) (Fig 2). We used a SureSelectXT solution-based target enrichment library preparation kit (Agilent Technologies) to create libraries for sequencing on the Illumina platform (conducted in 2011). RNA bait probes (120 bp) were designed to give 2X probe coverage of the 6.5 Mb target region using the online tool e-array (https://earray.chem.agilent.com/earray/). The total number of baits designed was 57,305, covering 3.8 Mb of the 6.5 Mb target region after repeat masking (58.5% base coverage). Genomic DNA (1.5 µg) from the five cases and five controls was used to prepare libraries for sequencing. DNA was Covaris fragmented at the Eastern Sequence and Informatics Hub, Cambridge, UK, followed by in-house library preparation. Paired-end sequencing (51 bp reads) was carried out on a single lane of an Illumina HiSeq2000 at the High-Throughput Genomics Group, Wellcome Trust Centre for Human Genetics, University of Oxford, UK, producing an 18 Gb dataset. The average read depth for the 10 libraries was 195X and the average percentage of target region coverage at 20X and 50X was 70% and 62%, respectively. Sequence reads for each dog were aligned to the canine reference genome (BROADD2) using BWA [25]. SNP/insertion-deletion (in-del) calls were made using GATK [26].

### Whole genome sequencing

WGS was done by Edinburgh Genomics, UK using TruSeq Nano library preparation and sequencing on an Illumina HiSeq X instrument at approximately 30X read depth. Paired-end sequence data were aligned to the CanFam 4 canine genome assembly using the BWA-MEM algorithm [25] and variant calls were made using GATK v3.6 [26]. WGS data were viewed using Integrative Genomics Viewer (IGV) software [11].

WGS of the Finnish Samoyed sample was done utilising an Illumina HiSeq2500 instrument with approximately 15X read depth and paired-end reads. All reads were aligned to the dog reference genome assembly CanFam 3.1 using BWA version 0.5.9 [25]. Picard tools (http://sourceforge.net/projects/picard) was used to mark duplicate reads and to sort the data. Local alignment, BAM file generation and variant calling were performed using GATK version 2.6 [26].

These analyses were conducted 2018–2024

### Analysis of variants

Potential susceptibility variants were genotyped using Taqman allelic discrimination assays. The association between SNPs and HC was tested using the Fisher’s exact test. LD among SNPs was assessed using pairwise correlation (r^2^).

### RNA sequencing

We obtained mRNA sequence for the *CPT1A* gene from a normal dog retina (Dalmatian breed) using mRNA-seq as described in [27]. Paired-end sequencing (81 bp reads) was carried out in-house on an Illumina MiSeq, producing a 4.3 Gb dataset. Reads were aligned to the canine reference genome (CanFam 3.1) using BWA [25]. Quality scores were re-calculated using GATK [26]. Aligned reads were viewed using IGV [11]. We also conducted a de-novo assembly of the mRNA-seq data, which allowed us to construct a full mRNA sequence for the *CPT1A* retinal transcript. Partial coding mRNA sequence was also obtained from a normal dog lens (Shar Pei) by PCR and Sanger sequencing using standard protocols. Primer sequences are given in S11 Table.

## Supporting information

S1 Fig Manhattan plot depicting the results of GWAS meta-analysis of the Samoyed and Siberian Husky. The analysis comprised 56 cases and 86 controls, 84,264 SNPs, top SNP BICF2P762157 at 53014634 bp, P = 3.0 x 10-6 (BROADD2 genome build; see S1 Table for LiftOver to other canine assemblies). The horizontal red dotted line denotes genome-wide statistical association (P <  5.9 x 10^-7^).
(PNG)

S2 Fig Manhattan plot depicting results of conditional GWAS analysis in the Siberian Husky in the discovery set (A) and replication set (B). Plot A: The analysis comprised 33 cases and 61 controls, 108,208 SNPs, with covariate adjustment for top GWAS SNP BICF2P1390488. Plot B: The analysis comprised 152 cases and 149 controls, 81,229 SNPs, with covariate adjustment for top GWAS SNP 18_49165418 (CanFam 3.1 assembly). The horizontal red dotted lines denote genome-wide statistical association (P <  4.6 x 10^-7^ and <  6.2 x 10^-7^ for (A) and (B) respectively). Plot (A) shows BROADD2 data (Illumina canineHD) and plot (B) shows array data mapped to the CanFam 3.1 genome assembly. See **S1 Table** for LiftOver to other assemblies.
(TIF)

S3 Fig DoGA expression atlas of CPT1A in canine tissues confirms its expression in ocular tissues and retina. (PNG)

S4 Fig Regional association plot of the genomic region surrounding the HC signal on CFA18 in the Siberian Husky replication set. The horizontal red dotted line denotes genome-wide statistical association (P <  4.6 x 10-7). The plot shows array data mapped to the CanFam 3.1 genome assembly. See **S1 Table** for LiftOver to other assemblies.
(PNG)

S5 Fig Regional association plot of the genomic region surrounding the HC signal on CFA18 in the Siberian Husky (blue diamonds) and Samoyed (grey diamonds). The horizontal red dotted line denotes genome-wide statistical association (P <  4.6 x 10-7), and the green line represents the empirical statistical threshold used to delineate the critical region in the Siberian Husky (P-value < 0.01). The vertical black dashed lines denote the start and end points of this critical region at chr18:51773736 bp and 58230092 bp (BROADD2 genome build; see **S1 Table** for LiftOver to other canine assemblies).
(PNG)

S1 Table LiftOver genomic positions of key variants and regions in the study.
Positions were also manually confirmed for correct genomic context between canine genome assemblies.
(XLSX)

S2 Table Genotypes for two top CFA18 SNP signals for HC in resequenced Siberian Huskies.
(DOCX)

S3 Table Association between SNP_57736166 and HC in Northern breeds.
(DOCX)

S4 Table Association between SNP_52196958 and HC in additional non-Northern breeds.
(DOCX)

S5 Table Association between SNP_52196196 and HC in Northern breeds.
(DOCX)

S6 Table Association between SNP_52193311 and HC in Northern breeds.
(DOCX)

S7 Table Association between SNP_52198711 and HC in Northern breeds.
(DOCX)

S8 Table Variants within 28 kb haplotype shared amongst five target-sequenced Husky cases and four case WGS (Husky, Malamute, Norwegian Buhund and Samoyed).
For clarity, variants are shown for all three canine genome assemblies utilised during this study. Non-risk allele refers to canine reference sequence (same for all builds) and wildtype to the reference sequence allele. Splice site predictions were compared between reference and variant sequences using http://www.fruitfly.org/seq_tools/splice.html
(XLSX)

S9 Table Association between ALX4 duplication and HC in Husky GWAS set.
The presence/absence of the duplication was assessed as described in [21].
(DOCX)

S10 Table Genomic inflation factors for GWAS analyses.
(DOCX)

S11 Table Primer sequences for amplification of partial coding CPT1A transcript in canine lens.
(DOCX)

S1 File Secondary analysis of variants in target sequenced region allowing one discordant case or control.
(PDF)
