# Supplementary figures and images for: Common variants in the CPT1A gene are associated with cataracts in Northern breeds of domestic dog
Source: PLoS One. 2025 Apr 4;20(4):e0320878. doi: 10.1371/journal.pone.0320878 (PMC11970653; doi:10.1371/journal.pone.0320878)

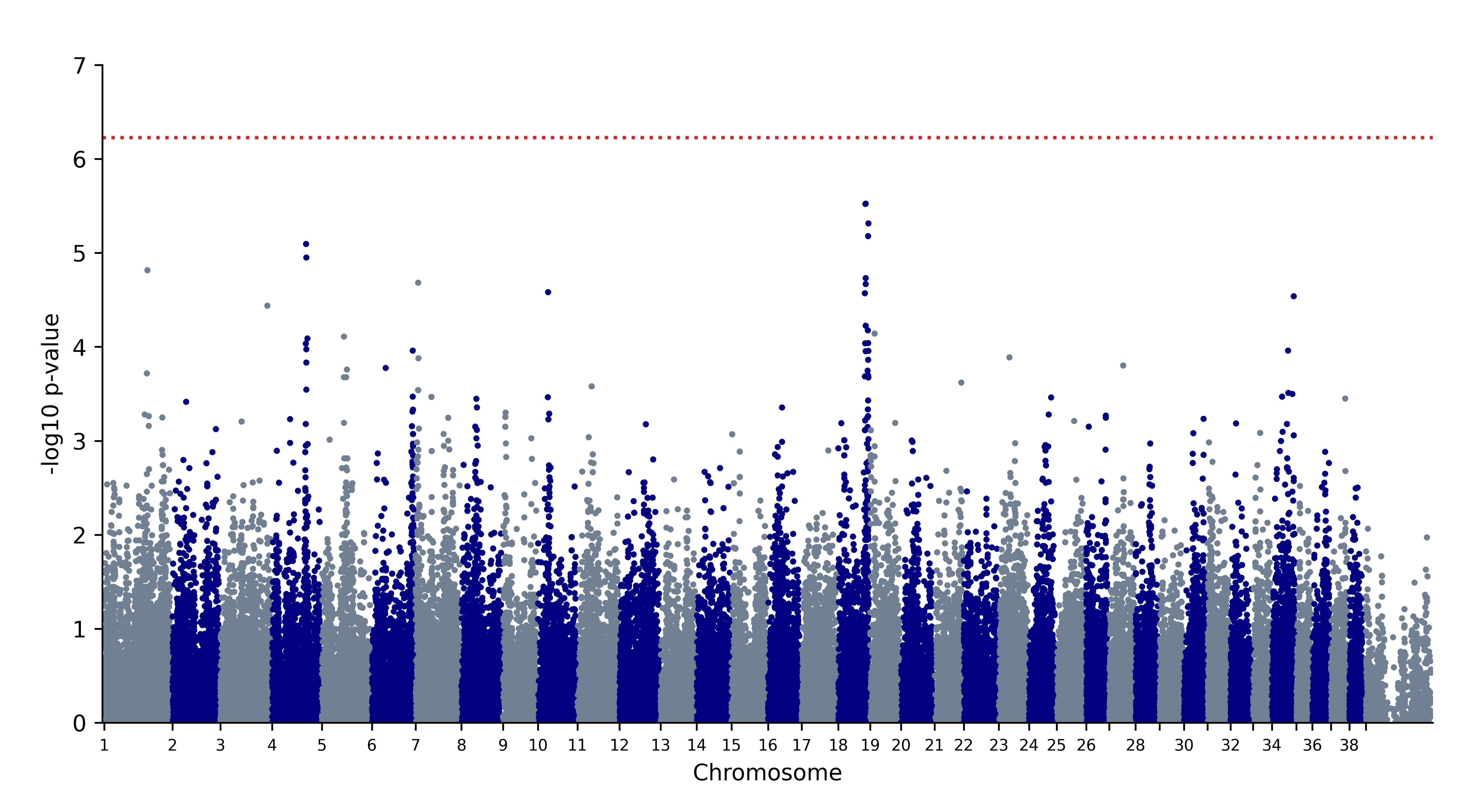

Supplement: S1 Fig — The analysis comprised 56 cases and 86 controls, 84,264 SNPs, top SNP BICF2P762157 at 53014634 bp, P = 3.0 x 10^-6^ (BROADD2 genome build; see S1 Table for LiftOver to other canine assemblies). The horizontal red dotted line denotes genome-wide statistical association (P < 5.9 x 10^-7^). (PNG) [file pone.0320878.s001.png]

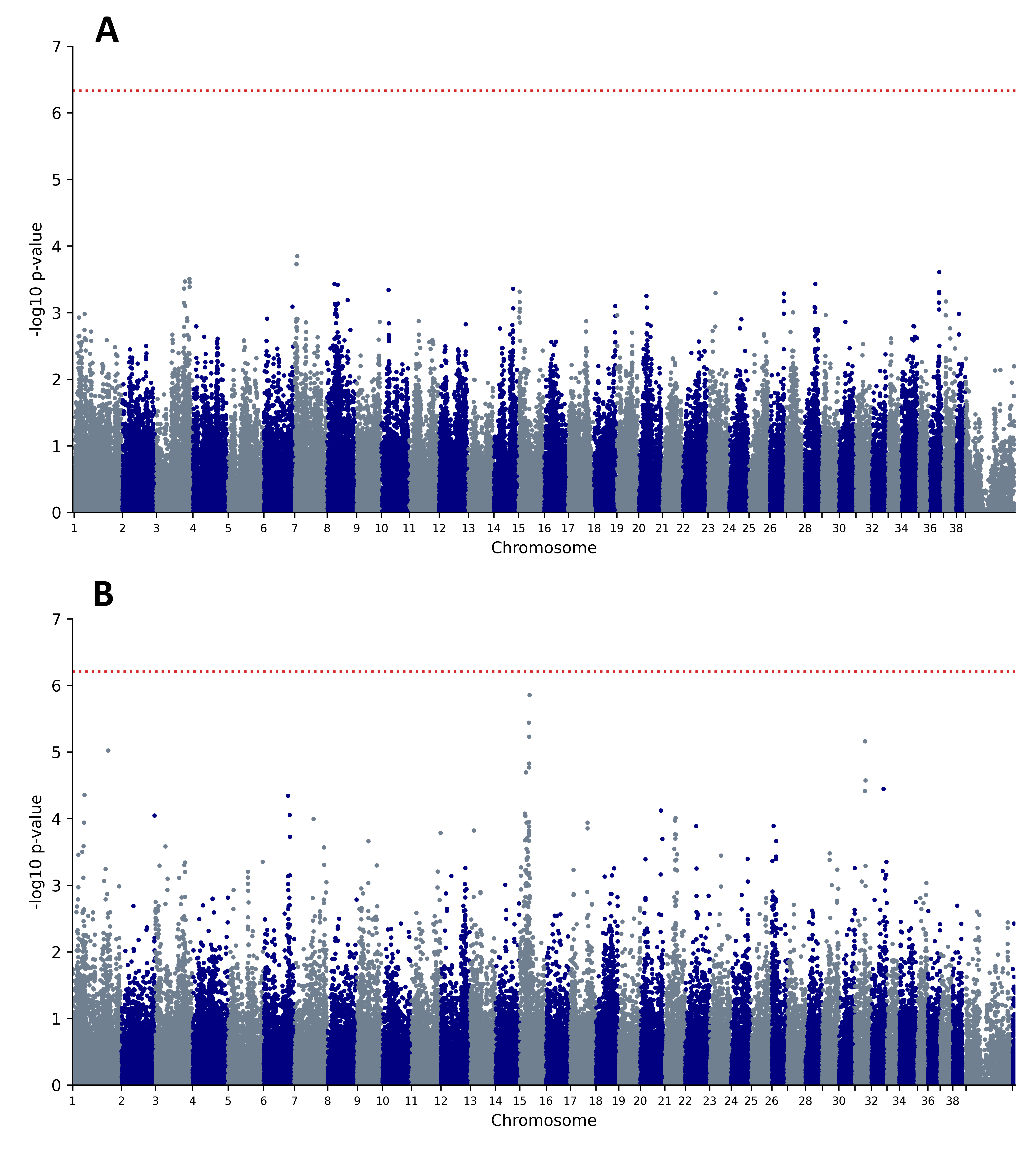

Supplement: S2 Fig — (A) and replication set (B). Plot A: The analysis comprised 33 cases and 61 controls, 108,208 SNPs, with covariate adjustment for top GWAS SNP BICF2P1390488. Plot B: The analysis comprised 152 cases and 149 controls, 81,229 SNPs, with covariate adjustment for top GWAS SNP 18_49165418 (CanFam 3.1 assembly). The horizontal red dotted lines denote genome-wide statistical association (P < 4.6 x 10^-7^ and < 6.2 x 10^-7^ for (A) and (B) respectively). Plot (A) shows BROADD2 data (Illumina canineHD) and plot (B) shows array data mapped to the CanFam 3.1 genome assembly. See S1 Table for LiftOver to other assemblies. (TIF) [file pone.0320878.s002.tif]

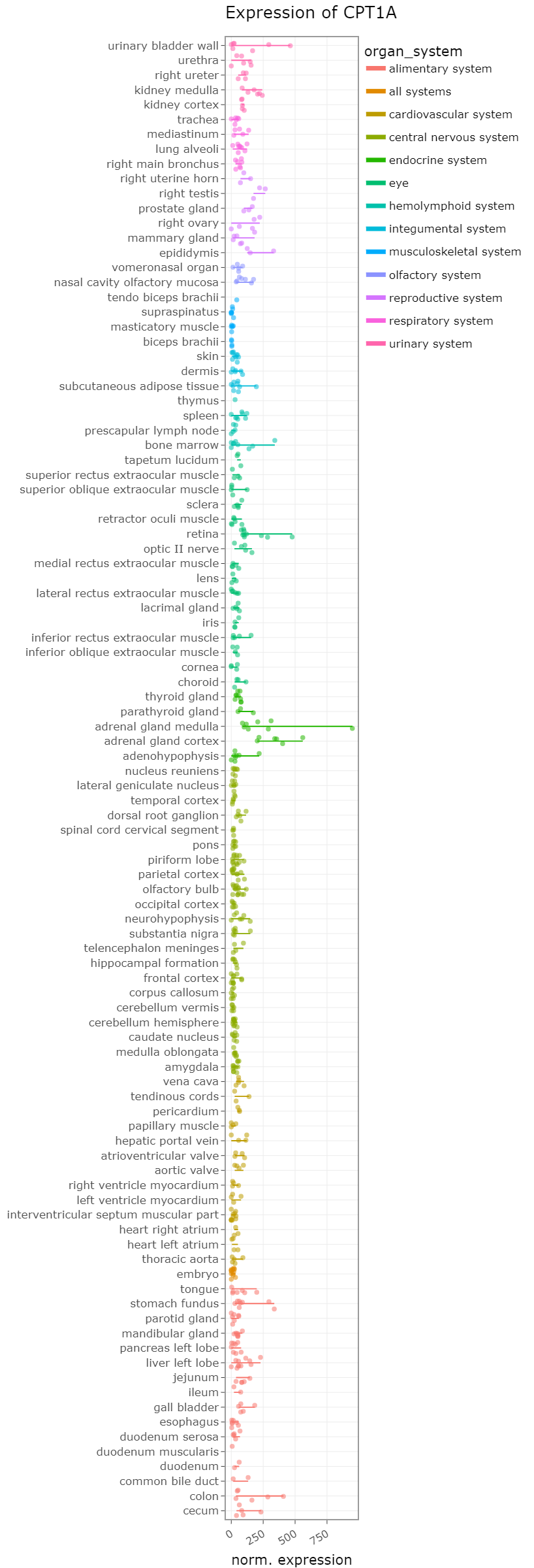

Supplement: S3 Fig — (PNG) [file pone.0320878.s003.png]

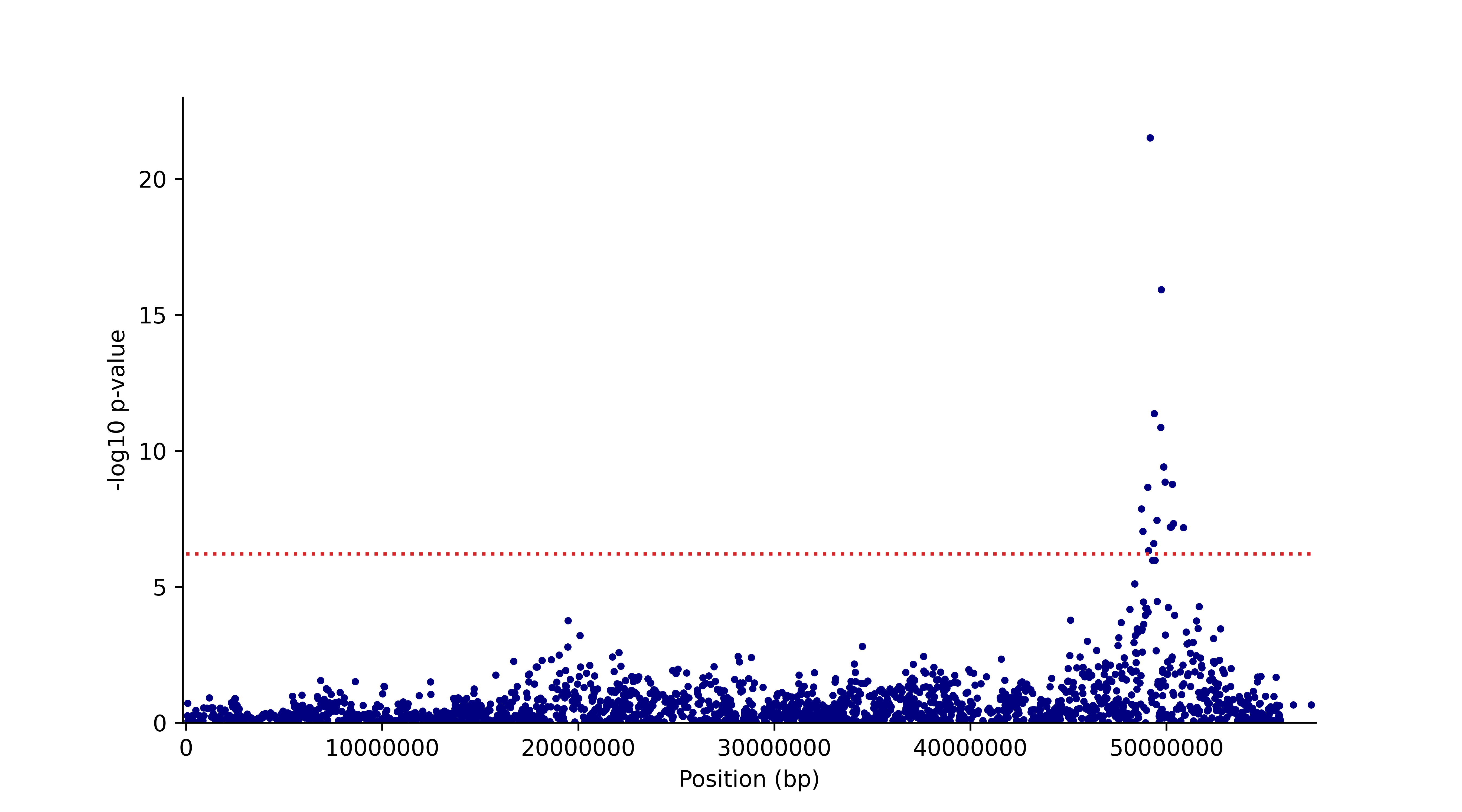

Supplement: S4 Fig — The horizontal red dotted line denotes genome-wide statistical association (P < 4.6 x 10^-7^). The plot shows array data mapped to the CanFam 3.1 genome assembly. See S1 Table for LiftOver to other assemblies. (PNG) [file pone.0320878.s004.png]

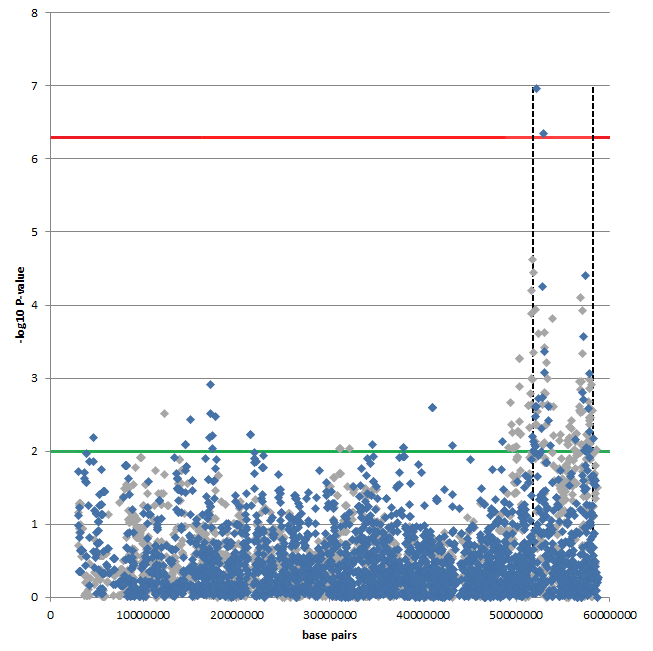

Supplement: S5 Fig — The horizontal red dotted line denotes genome-wide statistical association (P < 4.6 x 10^-7^), and the green line represents the empirical statistical threshold used to delineate the critical region in the Siberian Husky (P-value < 0.01). The vertical black dashed lines denote the start and end points of this critical region at chr18:51773736 bp and 58230092 bp (BROADD2 genome build; see S1 Table for LiftOver to other canine assemblies). (PNG) [file pone.0320878.s005.png]
