# Supplementary material for: Common variants in the CPT1A gene are associated with cataracts in Northern breeds of domestic dog
Source: PLoS One. 2025 Apr 4;20(4):e0320878. doi: 10.1371/journal.pone.0320878 (PMC11970653; doi:10.1371/journal.pone.0320878)
Supplement: S2 Table — (DOCX) [file pone.0320878.s007.docx]

| **Genotypes for two top CFA18 SNP signals for HC in resequenced Siberian Huskies** | | | |
| --- | --- | --- | --- |
| **Dog ID** | **Case/control status** | **SNP genotypes** | |
|  |  | **BICF2P1390488 (C/T)**  **52197069 bp**  **Risk allele: C** | **BICF2G630689379 (C/A)**  **57409134 bp**  **Risk allele: A** |
| 369 | Case | C/C | C/A |
| 371 | Case | C/C | A/A |
| 380 | Case | C/C | A/A |
| 386 | Case | C/C | A/A |
| 387 | Case | C/C | C/A |
| 367 | Control | C/T | C/C |
| 372 | Control | C/T | C/A |
| 377 | Control | C/T | C/C |
| 394 | Control | C/T | C/C |
| 396 | Control | C/T | C/C |
| BROADD2 genome build. See **S1 Table** for LiftOver of co-ordinates amongst canine genome assemblies. | | | |
