# Supplementary material for: Common variants in the CPT1A gene are associated with cataracts in Northern breeds of domestic dog
Source: PLoS One. 2025 Apr 4;20(4):e0320878. doi: 10.1371/journal.pone.0320878 (PMC11970653; doi:10.1371/journal.pone.0320878)
Supplement: S3 Table — (DOCX) [file pone.0320878.s008.docx]

| **Association between SNP_57736166 and HC in Northern breeds** | | | | | | | | |  |
| --- | --- | --- | --- | --- | --- | --- | --- | --- | --- |
|  |  |  |  | **Genotypes †**  **(cases/controls)** | | | **Allele frequencies †**  **(cases/controls)** | | **Fisher’s exact P-value** |
| **Breed** | **Case definition ‡** | **Control definition ∞** | **n cases/controls** | **GG** | **GT** | **TT** | **G** | **T** |  |
|  |  |  |  |  |  |  |  |  |  |
| Siberian Husky | OU PPSC | NAD | 43 / 130 | 22 / 91 | 15 / 35 | 6 / 4 | 0.69 / 0.83 | 0.31 / 0.17 | 0.01 |
| Siberian Husky | Other cataract | NAD | 17 / 130 | 11 / 91 | 5 / 35 | 1 / 4 | 0.79 / 0.83 | 0.21 / 0.17 | 0.52 |
|  |  |  |  |  |  |  |  |  |  |
| Samoyed | OU PPSC | NAD >=6 years of age | 30 / 81 | 8 / 42 | 14 / 32 | 8 / 7 | 0.50 / 0.72 | 0.50 / 0.28 | 0.02 |
| Samoyed | Other cataract | NAD >=6 years of age | 12 / 81 | 6 / 42 | 4 / 32 | 2 / 7 | 0.67 / 0.72 | 0.33 / 0.28 | 0.67 |
|  |  |  |  |  |  |  |  |  |  |
| **‡** OU PPSC: bilateral posterior polar subcapsular cataract; Other cataract: unilateral PPSC, cataract atypical for breed, e.g. nuclear, cortical, punctate cataract  ∞ NAD: no abnormality detected  **†** T = risk allele; G = non-risk allele (BROADD2 genome build. See **S1 Table** for LiftOver of co-ordinates amongst canine genome assemblies.) | | | | | | | | | |
