# Supplementary material for: Common variants in the CPT1A gene are associated with cataracts in Northern breeds of domestic dog
Source: PLoS One. 2025 Apr 4;20(4):e0320878. doi: 10.1371/journal.pone.0320878 (PMC11970653; doi:10.1371/journal.pone.0320878)
Supplement: S4 Table — (DOCX) [file pone.0320878.s009.docx]

| **Association between SNP_52196958 and HC in additional non-Northern breeds** | | | | | | | | |  |
| --- | --- | --- | --- | --- | --- | --- | --- | --- | --- |
|  |  |  |  | **Genotypes †**  **(cases/controls)** | | | **Allele frequencies †**  **(cases/controls)** | | **Fisher’s exact P-value** |
| **Breed** | **Case definition ‡** | **Control definition ∞** | **n cases/controls** | **GG** | **GC** | **CC** | **G** | **C** |  |
|  |  |  |  |  |  |  |  |  |  |
| Golden Retriever | OU PPSC | NAD >=6 years of age | 46 / 45 | 17 / 12 | 18 / 19 | 11 / 14 | 0.57 / 0.48 | 0.43 / 0.52 | 0.58 |
|  |  |  |  |  |  |  |  |  |  |
| Miniature Bull Terrier | Variable phenotype | ND | 10 / ND | 0 / ND | 3 / ND | 7 / ND | / ND | / ND | ND |
|  |  |  |  |  |  |  |  |  |  |
| **‡** OU PPSC: bilateral posterior polar subcapsular cataract; Other cataract: unilateral PPSC, cataract atypical for breed, e.g. nuclear, cortical, punctate cataract  ∞ NAD: no abnormality detected  **†** G = risk allele; C = non-risk allele (BROADD2 genome build. See **S1 Table** for LiftOver of co-ordinates amongst canine genome assemblies.)  ND: not done | | | | | | | | | |
