# Supplementary material for: Common variants in the CPT1A gene are associated with cataracts in Northern breeds of domestic dog
Source: PLoS One. 2025 Apr 4;20(4):e0320878. doi: 10.1371/journal.pone.0320878 (PMC11970653; doi:10.1371/journal.pone.0320878)
Supplement: S5 Table — (DOCX) [file pone.0320878.s010.docx]

| **Association between SNP_52196196 and HC in Northern breeds** | | | | | | | | |  |
| --- | --- | --- | --- | --- | --- | --- | --- | --- | --- |
|  |  |  |  | **Genotypes †**  **(cases/controls)** | | | **Allele frequencies †**  **(cases/controls)** | | **Fisher’s exact P-value** |
| **Breed** | **Case definition ‡** | **Control definition ∞** | **n cases/controls** | **TT** | **TC** | **CC** | **T** | **C** |  |
|  |  |  |  |  |  |  |  |  |  |
| Siberian Husky | OU PPSC | NAD | 43 / 138 | 41 / 55 | 2 / 72 | 0 / 11 | 0.98 / 0.66 | 0.02 / 0.34 | 6.3 x 10^-11^ |
| Siberian Husky | Other cataract | NAD | 18 / 138 | 14 / 55 | 3 / 72 | 1 / 11 | 0.86 / 0.66 | 0.14 / 0.34 | 6.9 x 10^-3^ |
|  |  |  |  |  |  |  |  |  |  |
| Samoyed | OU PPSC | NAD >=6 years of age | 30 / 83 | 17 / 10 | 7 / 35 | 6 / 38 | 0.68 / 0.33 | 0.32 / 0.67 | 1.4 x 10^-5^ |
| Samoyed | Other cataract | NAD >=6 years of age | 13 / 83 | 3 / 10 | 4 / 35 | 6 / 38 | 0.38 / 0.33 | 0.62 / 0.67 | 0.50 |
|  |  |  |  |  |  |  |  |  |  |
| Alaskan Malamute | OU PPSC | NAD | 46 / 120 | 39 / 92 | 7 / 26 | 0 / 2 | 0.92 / 0.88 | 0.08 / 0.13 | 0.49 |
| Alaskan Malamute | Other cataract | NAD | 19 / 120 | 16 / 92 | 3 / 26 | 0 / 2 | 0.92 / 0.88 | 0.08 / 0.13 | 0.82 |
|  |  |  |  |  |  |  |  |  |  |
| Icelandic Sheepdog | OU PPSC | NAD >=6 years of age | 12 / 35 | 12 / 9 | 0 / 17 | 0 / 9 | 1.00 / 0.50 | 0.00 / 0.50 | 3.0 x 10^-5^ |
|  |  |  |  |  |  |  |  |  |  |
| Finnish Lapphund | OU PPSC | NAD >=6 years of age | 27 / 81 | 4 / 7 | 10 / 46 | 13 / 28 | 0.33 / 0.37 | 0.67 / 0.63 | 0.17 |
|  |  |  |  |  |  |  |  |  |  |
| Lapponian Herder | OU PPSC | NAD >=6 years of age | 15 / 68 | 3 / 1 | 1 / 19 | 11 / 48 | 0.23 / 0.15 | 0.77 / 0.85 | 8.5 x 10^-3^ |
|  |  |  |  |  |  |  |  |  |  |
| **‡** OU PPSC: bilateral posterior polar subcapsular cataract; Other cataract: unilateral PPSC, cataract atypical for breed, e.g. nuclear, cortical, punctate cataract  ∞ NAD: no abnormality detected  **†** T = risk allele; C = non-risk allele (BROADD2 genome build. See **S1 Table** for LiftOver of co-ordinates amongst canine genome assemblies.) | | | | | | | | | |
