# Supplementary material for: Common variants in the CPT1A gene are associated with cataracts in Northern breeds of domestic dog
Source: PLoS One. 2025 Apr 4;20(4):e0320878. doi: 10.1371/journal.pone.0320878 (PMC11970653; doi:10.1371/journal.pone.0320878)
Supplement: S6 Table — (DOCX) [file pone.0320878.s011.docx]

| **Association between SNP_52193311 and HC in Northern breeds** | | | | | | | | |  |
| --- | --- | --- | --- | --- | --- | --- | --- | --- | --- |
|  |  |  |  | **Genotypes †**  **(cases/controls)** | | | **Allele frequencies †**  **(cases/controls)** | | **Fisher’s exact P-value** |
| **Breed** | **Case definition ‡** | **Control definition ∞** | **n cases/controls** | **CC** | **CT** | **TT** | **C** | **T** |  |
|  |  |  |  |  |  |  |  |  |  |
| Siberian Husky | OU PPSC | NAD | 43 / 138 | 41 / 56 | 2 / 74 | 0 / 8 | 0.98 / 0.67 | 0.02 / 0.33 | 1.1 x 10^-10^ |
| Siberian Husky | Other cataract | NAD | 18 / 138 | 14 / 56 | 3 / 74 | 1 / 8 | 0.86 / 0.67 | 0.14 / 0.33 | 6.8 x 10^-3^ |
|  |  |  |  |  |  |  |  |  |  |
| Samoyed | OU PPSC | NAD >=6 years of age | 30 / 83 | 24 / 56 | 6 / 26 | 0 / 1 | 0.90 / 0.83 | 0.10 / 0.17 | 0.45 |
| Samoyed | Other cataract | NAD >=6 years of age | 13 / 83 | 8 / 56 | 5 / 26 | 0 / 1 | 0.81 / 0.83 | 0.19 / 0.17 | 0.79 |
|  |  |  |  |  |  |  |  |  |  |
| Alaskan Malamute | OU PPSC | NAD | 46 / 120 | 39 / 92 | 7 / 26 | 0 / 2 | 0.92 / 0.88 | 0.08 / 0.13 | 0.49 |
| Alaskan Malamute | Other cataract | NAD | 19 / 120 | 16 / 92 | 3 / 26 | 0 / 2 | 0.92 / 0.88 | 0.08 / 0.13 | 0.82 |
|  |  |  |  |  |  |  |  |  |  |
| Icelandic Sheepdog | OU PPSC | NAD >=6 years of age | 12 / 35 | 12 / 10 | 0 / 16 | 0 / 9 | 1.00 / 0.51 | 0.00 / 0.49 | 3.6 x 10^-5^ |
|  |  |  |  |  |  |  |  |  |  |
| Finnish Lapphund | OU PPSC | NAD >=6 years of age | 27 / 81 | 7 / 11 | 8 / 46 | 12 / 24 | 0.41 / 0.42 | 0.59 / 0.58 | 0.04 |
|  |  |  |  |  |  |  |  |  |  |
| Lapponian Herder | OU PPSC | NAD >=6 years of age | 15 / 68 | 7 / 12 | 1 / 32 | 7 / 24 | 0.50 / 0.41 | 0.50 / 0.59 | 3.3 x 10^-3^ |
|  |  |  |  |  |  |  |  |  |  |
| **‡** OU PPSC: bilateral posterior polar subcapsular cataract; Other cataract: unilateral PPSC, cataract atypical for breed, e.g. nuclear, cortical, punctate cataract  ∞ NAD: no abnormality detected  **†** C = risk allele; T = non-risk allele (BROADD2 genome build. See **S1 Table** for LiftOver of co-ordinates amongst canine genome assemblies.) | | | | | | | | | |
