# Supplementary material for: Common variants in the CPT1A gene are associated with cataracts in Northern breeds of domestic dog
Source: PLoS One. 2025 Apr 4;20(4):e0320878. doi: 10.1371/journal.pone.0320878 (PMC11970653; doi:10.1371/journal.pone.0320878)
Supplement: S7 Table — (DOCX) [file pone.0320878.s012.docx]

| **Association between SNP_52198711 and HC in Northern breeds** | | | | | | | | |  |
| --- | --- | --- | --- | --- | --- | --- | --- | --- | --- |
|  |  |  |  | **Genotypes †**  **(cases/controls)** | | | **Allele frequencies †**  **(cases/controls)** | | **Fisher’s exact P-value** |
| **Breed** | **Case definition ‡** | **Control definition ∞** | **n cases/controls** | **CC** | **CT** | **TT** | **C** | **T** |  |
|  |  |  |  |  |  |  |  |  |  |
| Siberian Husky | OU PPSC | NAD | 27 / 109 | 27 / 44 | 0 / 56 | 0 / 9 | 1.00 / 0.66 | 0.00 / 0.34 | 1.1 x 10^-8 *^ |
| Siberian Husky | Other cataract | NAD | 17 / 109 | 13 / 44 | 3 / 56 | 1 / 9 | 0.85 / 0.66 | 0.15 / 0.34 | 0.01 |
|  |  |  |  |  |  |  |  |  |  |
| Alaskan Malamute | OU PPSC | NAD | 28 / 90 | 27/ 69 | 1 / 19 | 0 / 2 | 0.91 / 0.87 | 0.09 / 0.13 | 0.07 |
| Alaskan Malamute | Other cataract | NAD | 16 / 90 | 13 / 92 | 3 / 26 | 0 / 2 | 0.92 / 0.88 | 0.08 / 0.13 | 0.82 |
|  |  |  |  |  |  |  |  |  |  |
| Icelandic Sheepdog | OU PPSC | NAD >=6 years of age | 12 / 35 | 12 / 9 | 0 / 16 | 0 / 10 | 1.00 / 0.51 | 0.00 / 0.48 | 3.6 x 10^-5^ |
|  |  |  |  |  |  |  |  |  |  |
| Norwegian Buhund | OU PPSC | NAD >=4 years of age | 10 / 9 | 10 / 4 | 0 / 3 | 0 / 2 | 1.00 / 0.61 | 0.00 / 0.39 | 0.01 |
|  |  |  |  |  |  |  |  |  |  |
| **‡** OU PPSC: bilateral posterior polar subcapsular cataract; Other cataract: unilateral PPSC, cataract atypical for breed, e.g. nuclear, cortical, punctate cataract  ∞ NAD: no abnormality detected  **†** C = risk allele; T = non-risk allele (BROADD2 genome build. See **S1 Table** for LiftOver of co-ordinates amongst canine genome assemblies.)  ^*^ Comparing same case-control set of 27 cases and 109 controls Fisher’s exact P-value for SNP_52196958 was 9.7 x 10^-9^. | | | | | | | | | |
