# Supplementary material for: Common variants in the CPT1A gene are associated with cataracts in Northern breeds of domestic dog
Source: PLoS One. 2025 Apr 4;20(4):e0320878. doi: 10.1371/journal.pone.0320878 (PMC11970653; doi:10.1371/journal.pone.0320878)
Supplement: S9 Table — The presence/absence of the duplication was assessed as described in [21]. (DOCX) [file pone.0320878.s014.docx]

| **Association between ALX4 duplication and HC in a subset of dogs from the Siberian Husky GWAS set** | | | | | | |
| --- | --- | --- | --- | --- | --- | --- |
|  |  |  |  | **Genotypes**  **(cases/controls)** | | **1-sided Fisher’s exact P-value** |
| **Breed** | **Case definition ‡** | **Control definition ∞** | **n cases/controls** | **DUP** | **WT** |  |
|  |  |  |  |  |  |  |
| Siberian Husky | OU PPSC | NAD | 9 / 26 | 5 / 9 | 4 / 17 | 0.24 |
|  |  |  |  |  |  |  |
| ‡ OU PPSC: bilateral posterior polar subcapsular cataract  ∞ NAD: no abnormality detected  WT = wildtype | | | | | | |
