# Supplementary material for: Common variants in the CPT1A gene are associated with cataracts in Northern breeds of domestic dog
Source: PLoS One. 2025 Apr 4;20(4):e0320878. doi: 10.1371/journal.pone.0320878 (PMC11970653; doi:10.1371/journal.pone.0320878)
Supplement: S10 Table — (DOCX) [file pone.0320878.s015.docx]

| **Genomic inflation factors for GWAS analyses** | | |
| --- | --- | --- |
| **Breed** | **Genomic inflation factor (λ)** | |
|  | **Unadjusted** | **Adjusted for population effects using mixed model** |
| Siberian Husky | 1.09 | 1.04 |
| Siberian Husky replication set | - | 1.00 |
| Samoyed | 1.11 | 1.03 |
| Alaskan Malamute | 1.05 | 1.05 |
| Meta-analysis Siberian Husky and Samoyed | 1.06 | - |
| Conditional analysis in Siberian Husky | 1.16 | - |
| Conditional analysis in Siberian Husky replication set | - | 1.00 |
