# Supplementary material for: Common variants in the CPT1A gene are associated with cataracts in Northern breeds of domestic dog
Source: PLoS One. 2025 Apr 4;20(4):e0320878. doi: 10.1371/journal.pone.0320878 (PMC11970653; doi:10.1371/journal.pone.0320878)
Supplement: S11 Table — (DOCX) [file pone.0320878.s016.docx]

| **Primer sequences for amplification of partial coding *CPT1A* transcript in canine lens** | | |
| --- | --- | --- |
| **Primer name** | **Primer sequence (5’-3’)** | **Product size (bp)** |
| CPT1A_1F2 | GCCATGAAGCTCTTAAGCAAA | 1333 |
| CPT1A_1R | CCCATCTTCCCATTTTTGAA |  |
| CPT1A_2F | AAGTGTCGCCAAGCCTATTTT | 1012 |
| CPT1A_2R | TCTCTCCCACAAGGATGTACG |  |
