# Supplementary material for: Common variants in the CPT1A gene are associated with cataracts in Northern breeds of domestic dog
Source: PLoS One. 2025 Apr 4;20(4):e0320878. doi: 10.1371/journal.pone.0320878 (PMC11970653; doi:10.1371/journal.pone.0320878)
Supplement: S1 File — (PDF) [file pone.0320878.s017.pdf]

Secondary analysis of variants in target sequenced region allowing one discordant case or control

Variants segregating with disease status allowing one discordant case or control were identified and assessed (498 for the dominant model and 633 for the recessive model). These variants were checked for calling errors by manually viewing each aligned variant in IGV and variants where the putative risk allele was the same as the Boxer reference were excluded. This left 40 variants in or near 17 genes that were subsequently manually checked for species conservation and gene annotation by multi-species alignments and comparison with the CanFam 3.1 genome assembly annotation. We assessed the putative functional consequences of six SNPs in the following genes: *AHNAK* (3'UTR); *INCENP* (splice site); *IGHMBP2* (two nonsynonymous, one synonymous); and pyruvate carboxylase (*PC*) (nonsynonymous) using MutationTaster, SIFT and PolyPhen where appropriate. Of these, the non-risk allele (G) of the nonsynonymous SNP in *PC* (G>A chr18:53533360 BROADD2 exon 21; chr18:50507237 CanFam 3.1 exon 22) was conserved across 12 eutherian mammals and the risk allele (A) predicted to be disease-causing. This variant was re-genotyped in the ten target sequenced Huskies and subsequently genotyped and assessed for association with HC in 26 Husky cases and 101 controls; and 26 Malamute cases and 91 controls. The variant was not associated with HC in either breed (P-values for Fisher's exact test 0.38 and 0.47, respectively).
